# Clinical significance of robot-assisted laparoscopic surgery for rectal cancer: a retrospective propensity score matching analysis

**DOI:** 10.1007/s00423-025-03734-4

**Published:** 2025-05-21

**Authors:** Masayuki Ando, Takeru Matsuda, Kimihiro Yamashita, Hiroshi Hasegawa, Ryuichiro Sawada, Yasufumi Koterazawa, Naoki Urakawa, Hironobu Goto, Shingo Kanaji, Yoshihiro Kakeji

**Affiliations:** 1 Division of Gastrointestinal Surgery, Department of Surgery, Kobe University Graduate School of Medicine, Kobe, Japan; 2 Division of Minimally Invasive Surgery, Department of Surgery, Kobe University Graduate School of Medicine, 7-5-2 Kusunoki-chou, Chuo-ku, Kobe, 650-0017 Japan

**Keywords:** Rectal cancer, Robotic surgery, Laparoscopic surgery, Propensity score matching

## Abstract

**Purpose:**

Conventional laparoscopic surgery (CLS) for rectal cancer may sometimes be difficult. Robot-assisted laparoscopic surgery (RALS) is expected to overcome these technical challenges of CLS and provide better short-term outcomes. However, previous randomized controlled trials indicated that the safety and feasibility of RALS compared to CLS remain controversial; therefore, we assessed the safety and feasibility of RALS for rectal cancer compared with CLS.

**Methods:**

This study retrospectively reviewed 702 patients who had undergone anterior resection by CLS or RALS for rectal malignancies from January 2009 to December 2023. Among the patients, 313 and 75 were included in the CLS and RALS groups, respectively. Short- and midterm outcomes of the two groups were compared after performing propensity score matching analysis (PSM) to adjust for patient and tumor characteristics.

**Results:**

A total of 140 and 70 patients in the CLS and RALS groups, respectively, were matched using PSM. The bleeding amount and C-reactive protein (CRP) levels on postoperative days 1 and 3 were significantly lower, the operation time was longer, and the postoperative hospital stay was significantly shorter in the RALS group than in the CLS group. The Kaplan–Meier curves for cause-specific survival, relapse-free survival, and the cumulative incidence of local recurrence demonstrated no difference between the two groups.

**Conclusion:**

RALS for rectal cancer provided superior outcomes to CLS in terms of the bleeding amount, postoperative CRP levels, and postoperative hospital stay. The midterm oncological outcomes in RALS were comparable to those in CLS.

## Introduction

Conventional laparoscopic surgery (CLS) for rectal cancer has been introduced as minimally invasive surgery, exhibiting more favorable short-term outcomes, including reduced bleeding and complications, and earlier postoperative recovery than open surgery [1–3]. Moreover, two large randomized controlled trials (RCTs) revealed the noninferiority of CLS to open surgery in terms of oncological outcomes [4, 5]. However, other RCTs failed to demonstrate the noninferiority of CLS in successful resection and circumferential resection margin (CRM) negative rate, and a meta-analysis indicated that CLS for rectal cancer could increase the risk of unsuccessful resection [6–8]. Maintaining the quality of total mesorectal excision (TME) and CRM has been particularly challenging in CLS for patients with narrow pelvis or obesity [9–11]. Therefore, robot-assisted laparoscopic surgery (RALS) has been established to resolve the technical difficulties of CLS for rectal cancer.

RALS has several features lacking in CLS, including a stabilized three-dimensional camera, articulated functions, and motion scaling, which enable safer and more accurate surgical manipulation even in the narrow pelvis [12]. The REAL trial, an RCT conducted in China, revealed that RALS provided superior short-term results to CLS concerning blood loss, conversion rate, CRM positive rate, complications, and length of hospital stay [13]. However, two RCTs, the ROLLAR and COLLAR trials, failed to demonstrate the superiority of RALS over CLS in terms of short-term outcomes, indicating that the safety and feasibility of RALS remain controversial [14, 15]. Therefore, we investigated the safety and feasibility of RALS for rectal cancer compared with CLS in this study.

## Materials and methods

### Study design

This study included all patients with rectal malignancy who underwent anterior resection or Hartmann’s procedure from January 2009 to December 2023. The inclusion criteria for this study were patients with primary rectal malignancy including adenocarcinoma, squamous cell carcinoma, neuroendocrine tumor, or malignant melanoma, and those who underwent CLS or RALS. This study excluded patients with recurrent disease, multiple cancers, pathological T4b stage, and those who underwent transanal total mesorectal excision (ta-TME), inguinal lymph node dissection, and emergent surgery. Since the introduction of RALS at our institute in September 2019, RALS or CLS were determined according to the availability of robots at the institute, not according to the patient’s background or the difficulty of surgery. Patients’ profiles, surgical records, postoperative and pathological results, and prognosis data were reviewed using a database of rectal cancers that are prospectively managed at Kobe University. The Institutional Review Board and Ethics Committee of Kobe University Graduate School of Medicine (Institutional Review Board reference no.: B240182) approved this study.

This study was conducted following the STROBE guidelines.

### Perioperative management

The tumor stage was determined based on the Japanese Classification of Colorectal, Appendiceal, and Anal Carcinoma. This classification is primarily consistent with the Union for International Cancer Control TNM classification, except that lymph node metastasis in the inferior mesenteric artery and lateral region is defined as N3 and has generally been used in clinical practice in Japan. Patients whose tumors were below the peritoneal reflection and were diagnosed as cT3/4 or cN positive, with no distant metastases, received preoperative treatment including neoadjuvant chemoradiotherapy (NACRT) or neoadjuvant chemotherapy (NAC). NACRT consisted of the combination of oral 5-FU and 45–50 Gy of radiation therapy, followed by surgery 6–8 weeks after NACRT completion. NAC consisted of four cycles of FOLFOXIRI plus bevacizumab and the last two cycles of FOLFOXIRI without bevacizumab, followed by surgery 2–8 weeks after NAC. Patients who were pathologically diagnosed with stage III rectal cancer were considered for postoperative adjuvant chemotherapy such as oral or intravenous 5-FU and LV, CapeOX, or FOLFOX for 3–6 months. Postoperative surveillance was performed based on the Japanese Society for Cancer of the Colon and Rectum guidelines. Tumor markers were evaluated every 3 months, computed tomography (CT) every 6 months, and colonoscopy every year for the first 3 years postoperatively to detect recurrence or metastasis. Afterward, tumor markers and CT were assessed every 6 months.

### Surgical techniques

The da Vinci Xi surgical system (Intuitive Surgical, Sunnyvale, CA, USA) or the Hinotori surgical robot system (Medicaroid Corporation, Kobe, Hyogo, Japan) was used for RALS. RALS was performed as previously reported by Parascandola et al. [16]. In brief, the patient cart was rolled in after the four ports for the operator were placed in a straight line from the right lower quadrant to the left upper quadrant and two ports for the assistant in the right upper and middle quadrants. Inferior mesenteric vessel ligation, proximal lymphadenectomy, and descending and sigmoid colon mobilization were performed using a medial approach. The splenic flexure was mobilized if needed for anastomosis. TME or tumor-specific mesorectal excision was then completed 2–3 cm from the distal tumor margin, based on the tumor location. Anastomosis by the double stapling technique was performed in low anterior resection (LAR) and high anterior resection, whereas a colostomy was created in the Hartmann operation, after rectal transection using a linear stapler.

The same procedures described above were performed using laparoscopic devices in a standard 5-port fashion in CLS.

### Statistical analysis

We conducted a propensity score matching analysis (PSM) to minimize the bias due to the differences in patient and tumor characteristics between the two groups. A logistic regression model was utilized to calculate the propensity score using the following covariates: age, sex, American Society of Anesthesiology physical status classification, body mass index, distance from the anal verge, clinical T stage, surgical procedure, preoperative treatment, and lateral pelvic lymph node dissection. The patients in the CLS and RALS groups were finally matched and analyzed after nearest neighbor matching within 0.2 standard deviation of the caliper width so that the CLS group and RALS would be at a ratio of 2:1 without replacement. The standardized mean difference (SMD) was calculated to assess the difference in patient and tumor characteristics between the two groups before and after PSM, and an SMD value of < 0.1 was considered well-balanced. The chi-square or Fisher’s exact test was conducted to compare categorical variables, and the Mann–Whitney test was utilized to compare non-parametric continuous variables. Cause-specific survival (CSS), relapse-free survival (RFS), and the cumulative incidence of local recurrence (LR) were estimated with the Kaplan–Meier method, and the survival curves of the two surgical groups were compared using the log-rank test. A *P*-value of < 0.05 indicated statistical significance in this study.

EZR version 1.66 (Saitama Medical Center, Jichi Medical University, Saitama, Japan), which is a graphical user interface for R (The R Foundation for Statistical Computing, Vienna, Austria) was used for all statistical data analyses.

## Results

A total of 702 patients were retrospectively examined in this study. Meanwhile, patients who had undergone ta-TME, inguinal lymph node dissection, para-aortic lymphadenectomy, perineal reconstruction, emergency surgery, and other surgical procedures concurrently were excluded, as were patients with T4b tumors that had invaded adjacent organs, with multiple cancers, with recurrent tumor, with missing data, and other reasons. Hence, 313 and 75 patients in the CLS and RALS groups, respectively, were eligible for this study. After PSM, a total of 140 and 70 patients in the CLS and RALS groups, respectively, were finally matched and analyzed in this study.

Table 1 shows the patient and tumor characteristics. Significant differences were observed between the two groups in the proportion of tumor location and preoperative serum carcinoembryonic antigen levels before PSM. The SMD remained slightly higher than 0.1 in the proportion of tumor location, cT, cN, and cStage after PSM, but most of the patient and tumor characteristics were well-balanced between the two groups.

**Table 1:** Patient and tumor characteristics before and after PSM

	Before PSM				After PSM			
	CLS	RALS	*P*	SMD	CLS	RALS	*P*	SMD
	*n* = 313	*n* = 75			*n* = 140	*n* = 70		
Age *	69.00 (27.00, 96.00)	70.00 (41.00, 87.00)	0.43	0.107	68.00 (37.00, 96.00)	70.50 (41.00, 87.00)	0.876	0.059
**Sex**,** n (%)**			0.505	0.094			0.652	0.074
Male	202 (64.5)	45 (60.0)			89 (63.6)	42 (60.0)		
Female	111 (35.5)	30 (40.0)			51 (36.4)	28 (40.0)		
**BMI (kg/m** ^**2**^ ) *	22.10 (13.80, 43.00)	22.80 (16.20, 35.80)	0.203	0.109	22.75 (15.10, 42.00)	22.75 (16.20, 35.80)	0.977	0.009
**ASA score**,** n (%)**			0.594	0.13			0.92	0.058
I	33 (10.5)	10 (13.3)			17 (12.1)	8 (11.4)		
II	212 (67.7)	52 (69.3)			100 (71.4)	49 (70.0)		
III	68 (21.7)	13 (17.3)			23 (16.4)	13 (18.6)		
Distance from AV (cm) *	12.00 (0.00, 23.00)	10.00 (3.00, 20.00)	0.113	0.134	10.00 (2.00, 20.00)	10.00 (3.00, 20.00)	0.999	0.052
**Tumor location**,** n (%)**			< 0.001	0.602			0.435	0.196
RS	153 (48.9)	19 (25.3)			49 (35.0)	19 (27.1)		
Ra	96 (30.7)	43 (57.3)			65 (46.4)	39 (55.7)		
Rb	61 (19.5)	13 (17.3)			26 (18.6)	12 (17.1)		
P	3 (1.0)	0 (0.0)			0 (0.0)	0 (0.0)		
**Preoperative treatment**,** n (%)**			1	0.011			0.778	0.085
No	293 (93.6)	70 (93.3)			129 (92.1)	66 (94.3)		
Yes	20 (6.4)	5 (6.7)			11 (7.9)	4 (5.7)		
**cT**, n (%) **			0.456	0.198			0.814	0.142
1	74 (23.6)	18 (24.0)			28 (20.0)	18 (25.7)		
2	58 (18.5)	17 (22.7)			28 (20.0)	14 (20.0)		
3	144 (46.0)	28 (37.3)			62 (44.3)	28 (40.0)		
4	37 (11.8)	12 (16.0)			22 (15.7)	10 (14.3)		
**cN**, n (%) **			0.773	0.123			0.624	0.179
0	196 (62.6)	44 (58.7)			87 (62.1)	43 (61.4)		
1	80 (25.6)	20 (26.7)			42 (30.0)	18 (25.7)		
2	27 (8.6)	9 (12.0)			9 (6.4)	7 (10.0)		
3	10 (3.2)	2 (2.7)			2 (1.4)	2 (2.9)		
**cStage**, n (%) **			0.4	0.232			0.426	0.25
I	122 (39.0)	32 (42.7)			51 (36.4)	31 (44.3)		
II	66 (21.1)	10 (13.3)			33 (23.6)	10 (14.3)		
III	94 (30.0)	27 (36.0)			45 (32.1)	23 (32.9)		
IV	31 (9.9)	6 (8.0)			11 (7.9)	6 (8.6)		
CEA (ng/ml) *	3.60 (0.30, 562.40)	2.70 (0.60, 274.20)	0.024	0.096	3.60 (0.30, 336.30)	2.70 (0.60, 274.20)	0.07	0.035
CA19-9 (U/ml) *	13.00 (1.00, 2078.00)	13.00 (1.00, 927.00)	0.662	0.116	13.00 (1.00, 443.00)	13.50 (1.00, 927.00)	0.959	0.086

Abbreviations: PSM: propensity score matching, SMD: standardized mean difference, BMI: body mass index, ASA: American Society of Anesthesiologists, AV: anal verge

^*^ The data are expressed as the median (range)

^**^ Tumors were classified according to the Japanese Classification of Colorectal, Appendiceal, Anal Carcinoma

Table 2 summarizes the surgical outcomes. The proportion of surgical procedures was similar between the two groups. The median operation time was longer in the RALS group than in the CLS group; however, the bleeding amount was significantly less in the RALS group than in the CLS. No significant difference was observed, whereas the conversion rate in the RALS group was lower.

**Table 2:** Surgical outcomes after PSM

	CLS	RALS	*P*
	*n* = 140	*n* = 70	
**Surgical procedure**,** n (%)**			1
HAR	28 (20.0)	14 (20.0)	
LAR	104 (74.3)	52 (74.3)	
Hartmann	8 (5.7)	4 (5.7)	
**Lymphadenectomy**, n (%) *			0.005
prxD2	25 (17.9)	3 (4.3)	
prxD3	115 (82.1)	67 (95.7)	
**LLND**,** n (%)**			1
No	135 (96.4)	68 (97.1)	
Yes	5 (3.6)	2 (2.9)	
Operation time (min) **	286.00 (156.00, 720.00)	320.00 (215.00, 577.00)	0.003
The amount of bleeding (ml) **	0.00 (0.00, 600.00)	0.00 (0.00, 200.00)	< 0.001
**Transfusion**,** n (%)**			0.666
No	135 (96.4)	69 (98.6)	
Yes	5 (3.6)	1 (1.4)	
**Conversion**,** n (%)**			0.552
No	137 (97.9)	70 (100.0)	
Yes	3 (2.1)	0 (0.0)	
**Diverting ileostomy**,** n (%)**			0.818
No	115 (87.1)	59 (89.4)	
Yes	17 (12.9)	7 (10.6)	
The number of harvested LNs **	15.00 (3.00, 65.00)	15.00 (3.00, 34.00)	0.88

Abbreviations: PSM: propensity score matching, HAR: high anterior resection, LAR: low anterior resection, prx: proximal, LLND: lateral pelvic lymph node dissection, LN: lymph node

^*^ According to the Japanese Classification of Colorectal, Appendiceal, Anal Carcinoma

^**^ The data are expressed as the median (range)

Table 3 presents no significant difference between the two groups in the frequency of Clavien–Dindo classification grade I or higher complications. The median C-reactive protein (CRP) levels on postoperative days (PODs) 1 and 3 were significantly lower in the RALS group than in the CLS group. The median postoperative hospital stay was significantly shorter in the RALS group than in the CLS group (*P* = 0.006).

**Table 3:** Postoperative outcomes after PSM

	CLS	RALS	*P*
	*n* = 140	*n* = 70	
**Postoperative complications (CD II)**,** n (%)**	38 (27.1)	17 (24.3)	0.74
Urinary disturbance	3 (2.1)	4 (5.7)	
Anastomotic leakage	10 (7.1)	3 (4.3)	
Anastomotic bleeding	3 (2.1)	0 (0.0)	
Abdominal wound infection	1 (0.7)	3 (4.3)	
Bowel obstruction	4 (2.9)	3 (4.3)	
Paralytic ileus	1 (0.7)	1 (1.4)	
Lymphorrhea	1 (0.7)	0 (0.0)	
Bladder injury	0 (0.0)	0 (0.0)	
Ureteral injury	0 (0.0)	0 (0.0)	
DVT/PTE	0 (0.0)	0 (0.0)	
Pneumonia	2 (1.4)	1 (1.4)	
Others	15 (10.7)	6 (8.6)	
**Postoperative complications (CD III)**,** n (%)**	16 (11.4)	5 (7.1)	0.465
CRP levels on POD 1 (mg/dl) *	5.24 (0.66, 13.01)	4.00 (0.07, 11.42)	< 0.001
CRP levels on POD 3 (mg/dl) *	7.08 (0.77, 31.67)	5.49 (0.02, 16.74)	0.006
CRP levels on POD 7 (mg/dl) *	1.63 (0.00, 20.18)	1.23 (0.01, 20.89)	0.087
**Re-operation 30 days**,** n (%)**	11 (7.9)	3 (4.3)	0.394
**Mortality 30 days**,** n (%)**	0 (0.0)	0 (0.0)	1
Postoperative hospital stay (day) *	15.00 (9.00, 196.00)	12.50 (9.00, 90.00)	0.006

Abbreviations: PSM: propensity score matching, CD: Clavien-Dindo classification, DVT: deep vein thrombosis, PTE: pulmonary thromboembolism, CRP: C-reactive protein, POD: postoperative day

^*^ The data are expressed as the median (range)

Table 4 summarizes the pathological outcomes. A negative distal margin was achieved in all patients in this study, but a positive radial margin (RM) was confirmed only in one patient in the RALS group.

**Table 4:** Pathological outcomes after PSM

	CLS	RALS	*P*
	*n* = 140	*n* = 70	
**Histological type**,** n (%)**			0.497
tub1/tub2	132 (94.3)	66 (94.3)	
por/sig/muc	5 (3.6)	1 (1.4)	
others	3 (2.1)	3 (4.3)	
**pT**, n (%) *			0.942
0	5 (3.6)	2 (2.9)	
1	31 (22.1)	19 (27.1)	
2	19 (13.6)	10 (14.3)	
3	77 (55.0)	36 (51.4)	
4	8 (5.7)	3 (4.3)	
**pN**, n (%) *			0.685
0	87 (62.1)	47 (67.1)	
1	35 (25.0)	13 (18.6)	
2	17 (12.1)	10 (14.3)	
3	1 (0.7)	0 (0.0)	
**pStage**, n (%) *			0.826
0	5 (3.6)	1 (1.4)	
I	42 (30.0)	24 (34.3)	
II	37 (26.4)	19 (27.1)	
III	45 (32.1)	19 (27.1)	
IV	11 (7.9)	7 (10.0)	
**Lymphovascular invasion**,** n (%)**			0.877
Absent	46 (32.9)	24 (34.3)	
Present	94 (67.1)	46 (65.7)	
**DM involvement**,** n (%)**	0 (0.0)	0 (0.0)	1
**RM involvement**,** n (%)**	0 (0.0)	1 (1.4)	0.333

Abbreviations: PSM: propensity score matching, DM: distal margin, RM: radial margin

^*^ Tumors were classified according to the Japanese Classification of Colorectal, Appendiceal, Anal Carcinoma

**Fig. 1:**
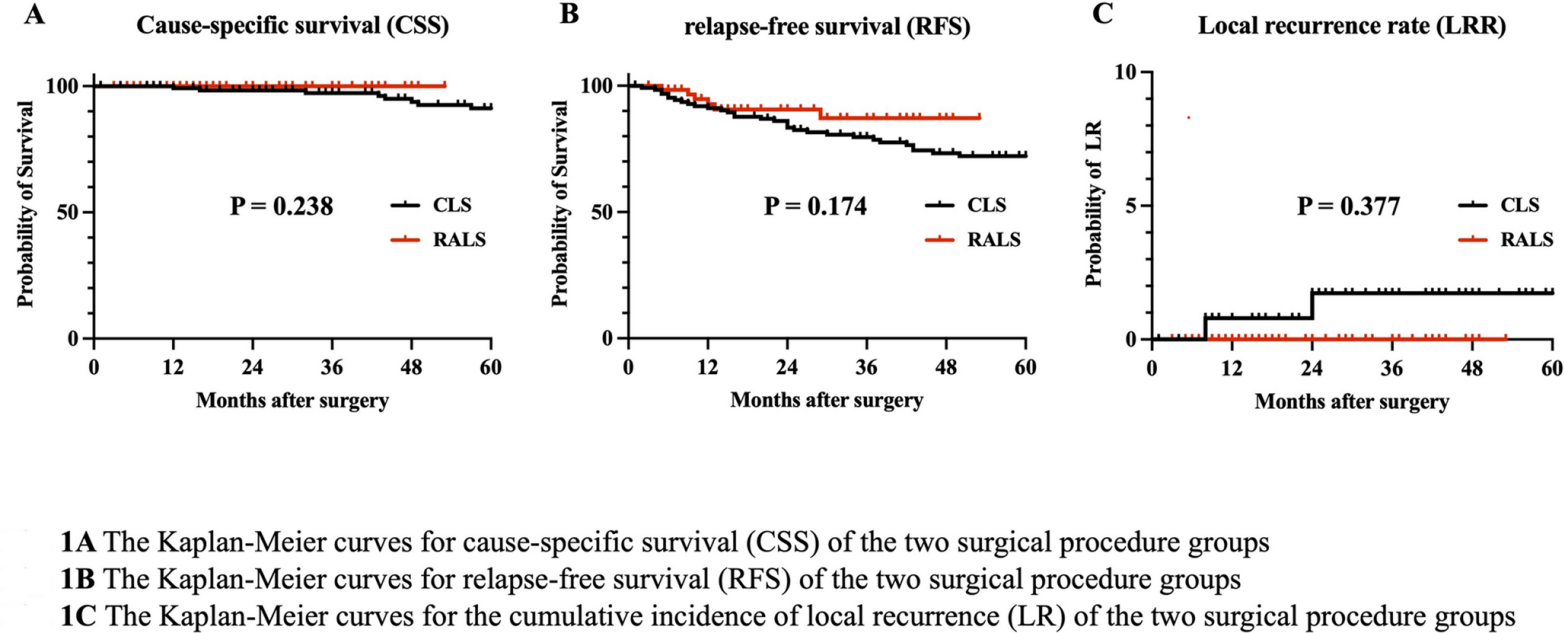
The Kaplan-Meier curves for cause-specific survival (**A**), relapse-free survival (**B**), and cumulative incidence of local recurrence (**C**) of the two surgical procedure groups

**Fig. 2:**
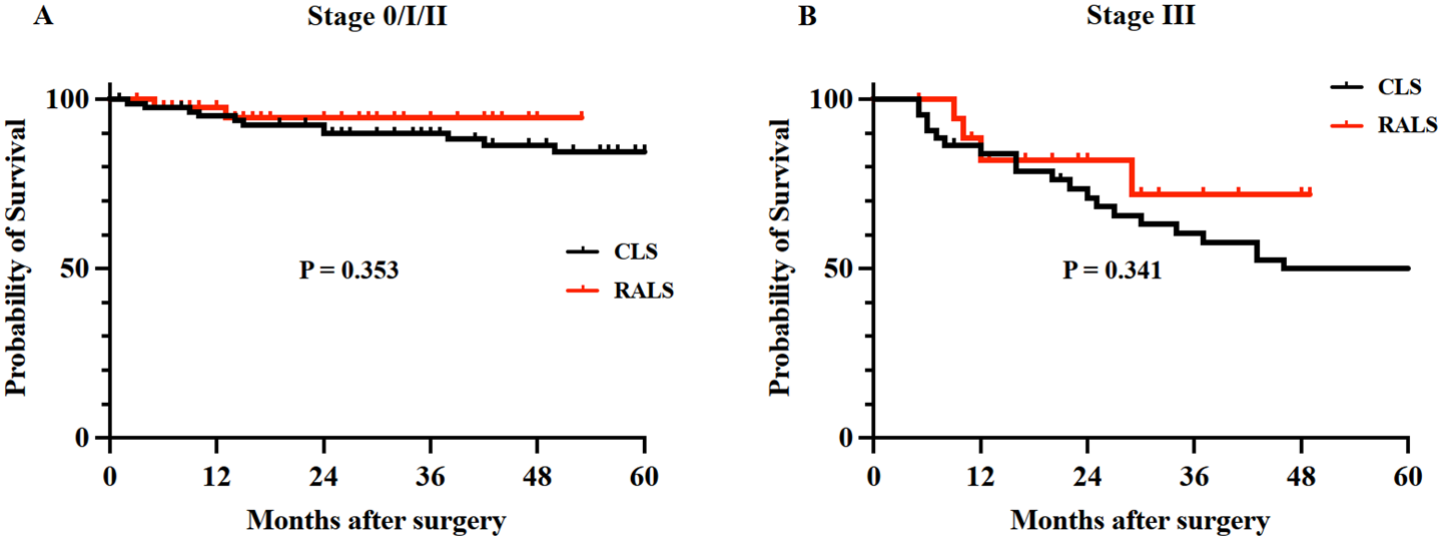
The Kaplan-Meier curves of the two surgical procedure groups in RFS for the pStage 0-II (**A**) and pStage III (**B**)

Figures 1 and 2 illustrate the midterm oncological outcomes. A total of 18 patients with stage III rectal cancer, consisting of 11 and 7 patients in the CLS and RALS groups, respectively, were excluded from the prognosis analysis. The median follow-up period was 60 months and 26 months in the CLS and RALS groups, respectively. The Kaplan–Meier curve for CSS demonstrated no significant difference, with 3-year CSS of 97.3% and 100% in the CLS and RALS groups, respectively (Fig. 1A).

The Kaplan–Meier curve for RFS indicated no significant difference between the CLS and RALS groups, with 3-year RFS of 79.7% and 87.2%, respectively (Fig. 1B). Further, no difference was observed in either stage 0–II or stage III concerning the Kaplan–Meier curve for RFS (Fig. 2). No difference in the cumulative incidence of LR was found between the CLS and RALS groups, with a 3-year LR of 1.7% and 0%, respectively (Fig. 1C).

## Discussion

RALS for rectal cancer has been rapidly expanding and is expected to overcome the technical challenges of CLS. However, previously conducted RCTs indicated that the safety and feasibility of RALS compared to CLS remain controversial, whereas previous retrospective studies reported the benefit of RALS concerning short-term outcomes [12–15, 17–19]. Therefore, we assessed the safety and feasibility of RALS in comparison with CLS in this study to confirm the genuine beneficial approach of RALS. We revealed that RALS significantly reduced bleeding amount and CRP levels on PODs 1 and 3 and shortened the postoperative hospital stay compared with CLS. Further, the midterm oncological outcomes, such as CSS, RFS, and the cumulative incidence of LR in RALS were as favorable as those in CLS.

The operation time was longer in the RALS group than in the CLS group in this study; however, the result of RALS needs to be considered to include the initial introduction period, that is, the learning phase. Several cases in the RALS group in the present study would be included in the learning phase, considering recent studies indicating that the learning phase in RALS could be overcome after 34–65 cases [16, 20, 21]. Meanwhile, a significant difference between the two groups in operation time may have occurred because the surgeons were more proficient in CLS. The operation time in RALS is expected to be shortened by overcoming the learning phase through the further accumulation of cases.

The bleeding amount and postoperative CRP levels were significantly less in the RALS group than in the CLS group in line with previous literature [22]. This might be because the improved visibility and ergonomics in the RALS group facilitated recognition of the correct layers and more delicate manipulation even in the narrow pelvic cavity. The delicate and minimal traction and reduced stress to the abdominal wall and peritoneum due to the pivot during RALS might also ultimately lead to less invasive and lower systemic inflammatory response compared to CLS. However, caution must be exercised in these results, because the incidence of the postoperative complications did not differ significantly between the groups. Further investigation would be necessary to clarify the clinical value and unexplored mechanisms of such differences.

Anastomosis leakage is a serious complication that affects short- and long-term outcomes in rectal cancer surgery. The frequency of anastomosis leakage was lower in RALS, with no significant difference in this study. This may be because RALS not only allows for delicate manipulation but also routinely evaluates blood flow in the reconstructed bowels using indocyanine green (ICG). This might contribute to the shorter postoperative hospital stay in RALS. The hospital stay was arguably somewhat longer than that in other studies. However, the postoperative hospital stay was set at 10–11 days in the clinical path in our hospital due to Japanese insurance system and there was no significant discrepancy.

LR is related to a poor prognosis in rectal cancer. The 3-year cumulative incidence of LR in the RALS group was 0% in this study, which was as favorable as the previously reported LR rate of 2.6–10.1% in CLS [1, 4, 5, 23]. This result may be because the better vision and ergonomics provided by RALS also improved the quality of surgery in terms of pathological results. The number of lymph nodes harvested in RALS was ≥ 12, and only one (2%) patient in RALS demonstrated a positive RM, which was comparable to the previous studies at 3–12.1% in CLS [4–7, 13–15, 24–26].

Furthermore, these favorable outcomes were achieved despite the presence of many male patients, those with locally advanced cancer, and those who had undergone LAR in the present study, thereby confirming the oncological safety of RALS. The CSS and RFS, in addition to LR, in RALS were as good as those in CLS in this study and comparable to those in previous literature [1, 4, 5, 23]. RFS analysis for each of stages 0–II and III demonstrated that RFS was consistently equivalent between the two groups, indicating that RALS may result in promising oncological outcomes, regardless of tumor progression such as early-stage or advanced. However, further investigation is warranted as the follow-up period for RALS was short.

This study has several limitations. First, the selection bias may remain in this retrospective study despite applying PSM to mitigate the bias. Second, the risk of overestimation exists regarding RM as the mesenteric lymph node is generally isolated from the specimen before the assessment in the pathology department in Japan, and the quality of TME has not been assessed. Third, because we started RALS since September 2019, many surgeries in RALS group were performed by surgeons in their learning phase for RALS. In contrast, CLS was introduced over 20 years ago and is now well standardized. Future studies of RALS performed by experienced surgeons might demonstrate stronger evidence to support a more definitive preference for RALS versus CLS. Finally, the recurrence and survival rate may be underestimated because the follow-up period for RALS remains short, thereby requiring further surveys.

## Conclusion

In conclusion, RALS significantly reduced the bleeding amount and postoperative CRP levels and shortened the postoperative hospital stay compared with CLS, and the midterm oncological outcomes in RALS were as favorable as those in CLS. Although further studies on a larger scale and over a longer period would be warranted, this study may support the feasibility and safety of RALS for rectal cancer.

## Data Availability

No datasets were generated or analysed during the current study.
